# High-Throughput Screening of Potential Skin Penetration-Enhancers Using Stratum Corneum Lipid Liposomes: Preliminary Evaluation for Different Concentrations of Ethanol

**DOI:** 10.1155/2017/7409420

**Published:** 2017-02-21

**Authors:** Pajaree Sakdiset, Yuki Kitao, Hiroaki Todo, Kenji Sugibayashi

**Affiliations:** ^1^Faculty of Pharmaceutical Sciences, Josai University, 1-1 Keyakidai, Sakado, Saitama 350-0295, Japan; ^2^School of Pharmacy, Walailak University, 222 Thai Buri, Tha Sala, Nakhon Si Thammarat 80160, Thailand

## Abstract

In this study, we developed a technique for high-throughput screening (HTS) of skin penetration-enhancers using stratum corneum lipid liposomes (SCLLs). A fluorescent marker, sodium fluorescein (FL), entrapped in SCLLs was prepared to provide a preliminary evaluation of the effect of different concentrations of ethanol on the disruption effect of SCLLs, which is an alternative for skin penetration-enhancing effects. In addition, SCLLs containing a fluorescent probe (DPH, TMA-DPH, or ANS) were also prepared and utilized to investigate SCLL fluidity. The results using SCLL-based techniques were compared with conventional skin permeation and skin impedance test using hairless rat skin. The obtained correlations were validated between FL leakage, SCLL fluidity with various probes, or skin impedance and increases in the skin permeation enhancement ratio (ER) of caffeine as a model penetrant. As a result, FL leakage and SCLL fluidity using ANS were considered to be good indices for the skin penetration-enhancing effect, suggesting that the action of ethanol on the SC lipid and penetration-enhancing is mainly on the polar head group of intercellular lipids. In addition, this screening method using SCLL could be utilized as an alternative HTS technique for conventional animal tests. Simultaneously, the method was found to be time-saving and sensitive compared with a direct assay using human and animal skins.

## 1. Introduction

Skin is of considerable interest as an administration route for drugs with both local and systemic effects. However, the main obstacle for drugs to pass through the skin and exert their pharmacological activities is the intrinsic barrier function of the stratum corneum (SC). The SC is the uppermost layer of the skin which consists of keratin-filled corneocytes and intercellular lipids [1, 2]. Generally, drugs in vehicles applied topically onto the skin firstly distribute into the SC and then diffuse mainly through the intercellular lipid domain [3]. Because the intercellular lipids organize into multilamellar complexes filling the intercellular spaces to provide a strong barrier against the entry of the exogenous drugs [4], approaches to overcome the skin barrier function have been investigated extensively to improve the skin permeability of drugs.

Use of chemical penetration enhancers (CPEs) is a conventional and effective approach to overcome the high barrier function of the SC. They promote the skin permeation of drugs by reducing skin barrier resistance [5]. CPEs may disrupt intercellular lipids or interact with intracellular proteins or with both regions in the SC [6]. Conventional CPEs have been screened using skin permeation experiments to determine the cumulative amount of skin permeation, flux, and permeability coefficient of drugs in the presence and absence of CPEs [7]. However, these conventional approaches are time-consuming, complicated, and resource-expensive. These methods are not practical for high-throughput screening (HTS) with various types and concentrations of CPEs as part of their discovery and development. In addition, increasing awareness of animal welfare issues nowadays has brought about a reduction in animal-based experiments, especially in the cosmetics industry. This situation gives rise to a need to develop an effective replacement approach that can provide HTS for effective CPEs and avoid the use of experimental animals.

SC lipid liposomes (SCLLs) consist of a lipid mixture similar to multilamellar lipid bilayers that are composed of ceramides, cholesterol, cholesterol esters, and fatty acids [4, 8]. SCLLs have been used as an SC intercellular lipid mimicking model to investigate the effect of agents on the skin permeability and the mechanism of enhanced skin delivery [9, 10]. One of the determination procedures is based on the change in transition temperature of SCLLs due to the chemicals determined by DSC [9]. However, this technique is also time-consuming and cannot simultaneously determine various samples, and it is difficult to obtain quantitative data. SCLLs containing entrapped marker(s) (e.g., glucose, mannitol, and calcein) have been applied to determine the release rate of the markers [9, 11–13]. In addition, labeled fluorescent probes are also used to measure the fluidizing effect of CPEs on SCLLs [14]. However, these methods are still not appropriate for HTS of CPEs because of the complicated analytical methods and rather long experimental time.

Thus, the aim of the present study was to establish a simple, quantitative, fast, and practical approach for HTS of potential CPEs. Ethanol was used as a model CPE to firstly investigate the potential of SCLLs based on the HTS methodology, because it is well known and is already in use to increase the skin penetration of various drugs such as hydrocortisone, 5-fluorouracil, and estradiol [15–17]. SCLLs entrapping a hydrophilic fluorescent probe, sodium fluorescein (FL), and incubating with three fluorescent probes with different lipophilicities were used to observe SCLL leakage and fluidity, respectively, and compared with conventional* in vitro* skin permeation tests as well as skin impedance test to determine the effectiveness of this HTS technology.

## 2. Materials and Methods

### 2.1. Materials

FL, lignoceric acid, palmitic acid, boric acid, potassium chloride, sodium hydroxide, chloroform, methanol, and ethanol were purchased from Wako Pure Chemicals Industries, Ltd. (Osaka, Japan). Cholesterol, cholesteryl sulfate, octacosanoic acid, 1,6-diphenyl-1,3,5-hexatriene (DPH), N,N,N-trimethyl-4-(6-phenyl-1,3,5-hexatrien-1-yl)phenyl-ammonium* p*-toluenesulfonate (TMA-DPH), and 8-anilino-1-naphthalenesulfonic acid ammonium salt (ANS) were from Sigma-Aldrich (St. Louis, MO, USA). Ceramide type III and ceramide type VI were from Evonik Industries AG (Essen, Germany). These reagents were used without further purification.

### 2.2. Experimental Animals

Male WBN/ILA-Ht hairless rats, weighing between 200 and 260 g, were obtained from the Life Science Research Center, Josai University (Saitama, Japan), and Ishikawa Experimental Animal Laboratories (Saitama, Japan). Rats were bred in a room maintained at 25 ± 2°C, in which the on and off times for the lighting were 07:00 and 19:00, respectively. Animals had free access to water and food (MF, Oriental Yeast Co., Ltd., Tokyo, Japan).

All breeding procedures and experiments on the animals were performed in accordance with the guidelines of the Animal Experiment Committee of Josai University.

### 2.3. Methods

#### 2.3.1. Preparation of Stratum Corneum Lipid Liposomes

SCLLs were prepared by a thin film hydration method reported by Hatfield and Fung [18] with a slight modification. The SCLLs had 5.5 mg/mL of total lipids including 33% ceramide type III, 22% ceramide type VI, 25% cholesterol, 5% cholesteryl sulfate, 7.5% lignoceric acid, 3.75% palmitic acid, and 3.75% octacosanoic acid. FL was used as an entrapped agent, and 0.1 M borate buffer (pH 9.0) was used as a dispersing medium. First, all lipid components were dissolved in the mixture of chloroform : methanol (2 : 1) in a round-bottomed flask. The solvent was then evaporated at 60°C under reduced pressure using a rotary evaporator until the thin film was obtained on the flask wall. The flask was then purged with N_2_ gas and allowed to stand overnight. Next, the flask was placed in a water bath at 90°C for 30 min for annealing of the thin lipid film. Then, 2.5 mg/mL FL in 0.1 M borate buffer was added to the flask and vortexed until the thin lipid film was completely dissolved. An ultrasonic probe (VCX-750, Sonics & Materials Inc., Newtown, CT, USA) was then immersed in the liposome suspension at an amplitude of 20% for 30 s. A freeze-thaw process was then performed by immersing the flask in liquid N_2_ followed by a 90°C water bath, each for 3 min for 4 cycles. The obtained liposomes were further extruded with a Lipex™ Extruder (Northern Lipids Inc., Burnaby, BC, Canada) using a membrane filter with pore sizes of 400, 200, and 100 nm (Nuclepore® track-etched membranes, GE Healthcare, Tokyo, Japan) (twice for each size of membrane filter). After preparation of the liposomes, they were centrifuged in an ultracentrifuge (Hitachi CS100GXL, Hitachi Koki Co., Ltd., Tokyo, Japan) at 289,000 ×g, 4°C twice for 15 min, twice for 10 min, and 5 times for 5 min. At each centrifugation process, the supernatant was removed and the same volume of 0.1 M borate buffer was added and mixed to prepare test liposome suspensions with removed free FL. The blank SCLLs were also prepared by previously explained process, without adding FL.

#### 2.3.2. Determination of Sodium Fluorescein Leakage from Stratum Corneum Lipid Liposomes

SCLLs containing FL (FL-SCLLs) and different concentrations of ethanol (0–100% ethanol in 0.1 M borate buffer) were placed at a ratio of 1 : 9 v/v in an ultracentrifuge tube and mixed 5 times by pipetting. The samples were allowed to stand for 30 min and immediately ultracentrifuged at 289,000 ×g, 4°C for 5 min. The supernatant was collected to determine the FL content using a microplate reader (SpectraMax® M2^e^, Molecular Devices, LLC., Sunnyvale, CA, USA) at excitation and emission wavelengths of 485 and 535 nm, respectively. This study was also performed using 75% ethanol and 0.1 M borate buffer to represent the total and background FL leakage, respectively. The FL leakage from SCLLs was then calculated using the following equation:

(1) $$\begin{array}{c} \text{FL leakage}\;\left(\;\%\;\right)=\left(\;\frac{{FL}_{\text{sample}}-\;{FL}_{\text{background}}}{{FL}_{\text{total}}-{FL}_{\text{background}}}\;\right)\times 100. \end{array}$$

#### 2.3.3. Measurement of Stratum Corneum Lipid Liposome Membrane Fluidity

The fluorescent probes DPH, TMA-DPH, and ANS were solubilized in phosphate-buffered saline (PBS) at concentrations of 2 × 10^−5^, 1 × 10^−5^, and 6 × 10^−3^ M, respectively. The fluidity of SCLL membranes was determined by incubating blank SCLLs and each fluorescent solution (25 : 75 *μ*L) in a 96-well plate and then shaking using an orbital shaker (IKA® MS 1 Minishaker, Sigma-Aldrich, Wilmington, NC, USA) at 500 rpm for 30 min in the absence of light. Next, different concentrations of ethanol in 0.1 M borate buffer (100 *μ*L) were added and shaken in the same conditions. The fluorescence polarization (*P*_*f*_) was measured at 25°C using a microplate reader (SpectraMax M5^e^, Molecular Devices, LLC., Sunnyvale, CA, USA). The respective excitation and emission wavelengths were 358 nm and 425 nm for DPH, 365 nm and 430 nm for TMA-DPH, and 337 nm and 480 nm for ANS. *P*_*f*_ value was calculated using the following equation:

(2) $$\begin{array}{c} P_{f}=\left(\;\frac{I_{0,0}-{GI}_{0,90}}{I_{0,0}+{GI}_{0,90}}\;\right), \end{array}$$

where *G* was the grating correction coefficient; *G* = *I*_90,0_/*I*_90,90_. In addition, the decrease in *P*_*f*_ indicating the degree of membrane fluidity was calculated using the following equation.

(3) $$\begin{array}{c} \text{Decrease in }P_{f}=\left(\;\frac{P_{f,\text{control}}-P_{f,\text{sample}}}{P_{f,\text{control}}}\;\right)\times 100, \end{array}$$

where *P*_*f*,control_ was obtained from blank SCLLs incubated with fluorescent probe and 0.1 M borate buffer.

#### 2.3.4. Measurement of Skin Impedance Reduction Rate

The mixture of three types of anesthesia (medetomidine, 0.375 mg/kg; butorphanol, 2.5 mg/kg; and midazolam, 2 mg/kg) was injected intraperitoneally into hairless rats. Hairs were removed from the back under anesthesia. The treated back skin was excised, and subcutaneous fat was removed carefully using scissors. The skin was mounted with the SC side up on a square-type diffusion cell with an effective area of 19.5 cm^2^ and a receiver volume of 45 mL. Eight donor cells with an effective diffusion area of 0.795 cm^2^ were glued with cyanoacrylate bond (Aron Alpha®, Konishi Co. Ltd., Osaka, Japan) onto the skin. Then, 1.0 and 45 mL of physiological saline were added to the donor and receiver cells, respectively, to hydrate the skin. Skin impedance was then determined using an impedance meter (Asahi Techno Lab., Ltd., Kanagawa, Japan) 1 h after hydration. Thereafter, the physiological saline was removed from the donor cell and 1.0 mL of different concentrations of ethanol was applied for 1 h. At the end of the experiment, the donor solution was removed, the same volume of fresh saline was added, and skin impedance was determined again. The skin impedance reduction (%) was calculated from the following equation.

(4) Skin  impedance  reduction %=1−Impedance  after  ethanol  pretreatmentImpedance  after  hydration×100.

#### 2.3.5. *In Vitro* Skin Permeation Experiments

Excised abdominal skin from hairless rat was mounted in a vertical-type Franz diffusion cell (receiver cell volume is 6.0 mL and effective permeation area is 1.77 cm^2^) with the SC side facing the donor cells and the dermal side facing the receiver cell. The receiver cell was filled with 6.0 mL PBS (pH 7.4). PBS was also added to the donor cells for 1 h for skin hydration. Then, 0.5 mL of 2 mg/mL caffeine solution in 0, 10, 20, 30, 40, 50, 75, or 100% ethanol in PBS was applied to determine the skin permeation of caffeine at 32°C over 8 h, while the receiver solution was agitated at 500 rpm using a magnetic stirrer. At predetermined times, 0.5 mL aliquots were collected and the same volume of PBS was added to keep the volume constant.

#### 2.3.6. Determination of Caffeine Permeation

The concentration of caffeine was determined using a high-performance liquid chromatography (HPLC) system (Prominence, Shimadzu Corporation, Kyoto, Japan) equipped with a UV detector (SPD-M20A, Shimadzu Corporation). Briefly, the receiving solutions were mixed with the same volume of methanol. After centrifugation at 21,500 ×g at 4°C for 5 min, the resulting supernatant (20 *μ*L) was injected directly into the HPLC system. Chromatographic separation was performed at 40°C using an Inertsil ODS-3, 5 *μ*m in diameter, 4.6 mm I.D. × 150 mm (GL Sciences Inc., Tokyo, Japan). The mobile phase was 0.1% phosphoric acid : methanol 7 : 3 v/v and the flow rate was 1.0 mL/min. UV absorbance detection was performed at 280 nm.

#### 2.3.7. Calculation of Skin Permeation Parameter

Skin permeation flux of 5 to 8 h after starting the experiment was calculated by linear regression of the skin permeation profile of caffeine. Skin permeation coefficient was obtained by dividing the skin permeation flux with the initial concentration of the applied caffeine in the donor compartment. The increase in the skin-penetration-enhancement ratio (ER) after an 8 h permeation experiment was calculated with a following equation.

(5) $$\begin{array}{c} \text{Increase in ER}=\left(\;\frac{Q_{\text{sample}}-Q_{\text{control}}}{Q_{\text{control}}}\;\right), \end{array}$$

where *Q*_sample_ and *Q*_control_ are the cumulative amounts of caffeine permeated per unit area of skin over 8 h from different concentrations of ethanol and PBS, respectively.

#### 2.3.8. Statistics

The differences among the obtained data were analyzed using unpaired *t*-test. The differences were considered significant when *p* < 0.05. Pearson's correlation coefficient was used to determine the relationship between the results from SCLL test and skin permeation study.

## 3. Results and Discussion

### 3.1. Results

#### 3.1.1. Effect of Ethanol on* In Vitro* Skin Permeation of Caffeine

**Figure 1:**
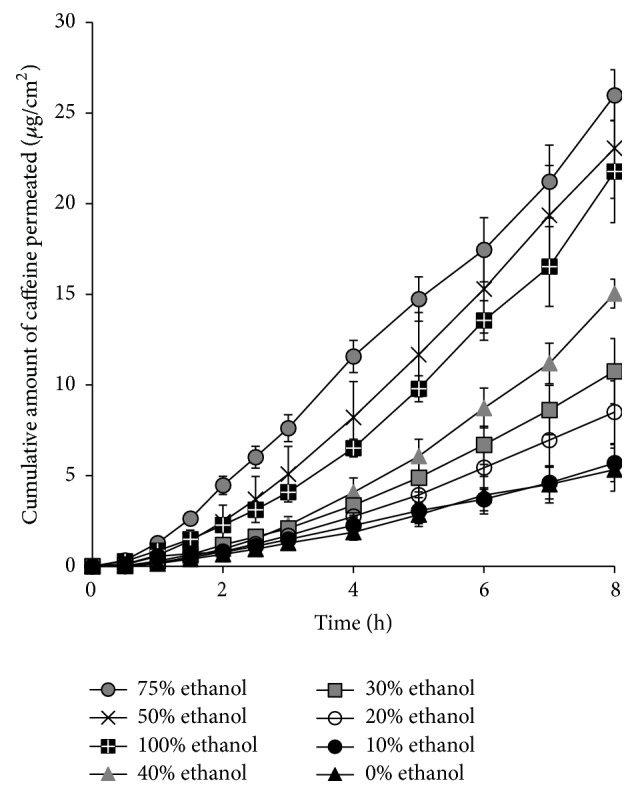
Time course of cumulative amount of caffeine permeated through full-thickness hairless rat skin after application with different concentrations of ethanol. Each value represents the mean ± SE (*n* = 3–5).

**Figure 2:**
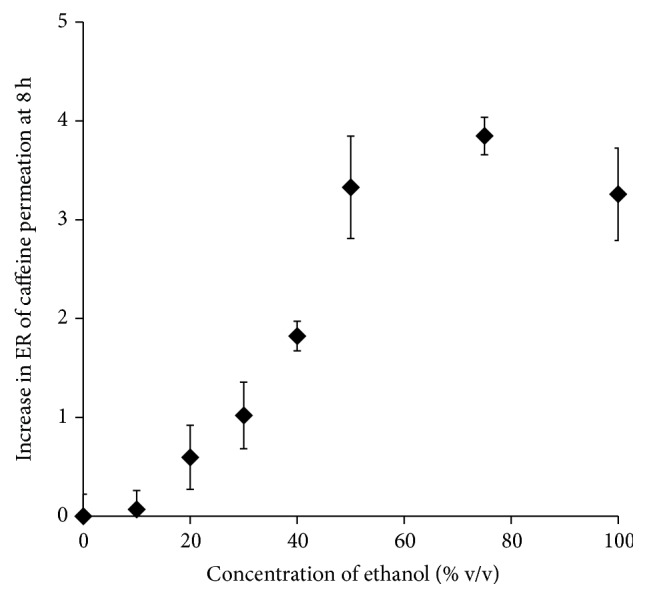
Effect of ethanol concentration on the increase in the skin-penetration-enhancement ratio (ER) of caffeine permeated over 8 h. Each value represents the mean ± SE (*n* = 3–5).

In the present study, caffeine was selected as a hydrophilic model drug to evaluate the relationship between its skin permeation rate and the effect of different concentrations of ethanol on SC lipid disruption.  Figure 1 shows the cumulative amounts of caffeine permeated through the excised hairless rat skin. Typical* in vitro* skin permeation profiles of caffeine (lag time and following almost steady-state profiles) were observed in all cases when using 0–100% ethanol as in the donor solution.  Figure 2 illustrates the relationship between the increase in ER and the ethanol concentration in the donor solution. Figures 1 and 2 show that the skin permeation of caffeine was strongly dependent on the ethanol concentration in the caffeine solution. When the ethanol concentration increased from 0% to 50%, the skin permeation rate of caffeine also increased. However, at 100% ethanol, caffeine permeation was rather decreased compared with the samples with 50% and 75% ethanol. The maximum skin permeation of caffeine was observed with 75% ethanol in the present study. The skin permeation flux, permeability coefficient, and enhancement ratio of caffeine obtained from different concentrations of ethanol are also summarized in Table 1.

#### 3.1.2. Effect of Ethanol on the Skin Impedance

**Figure 3:**
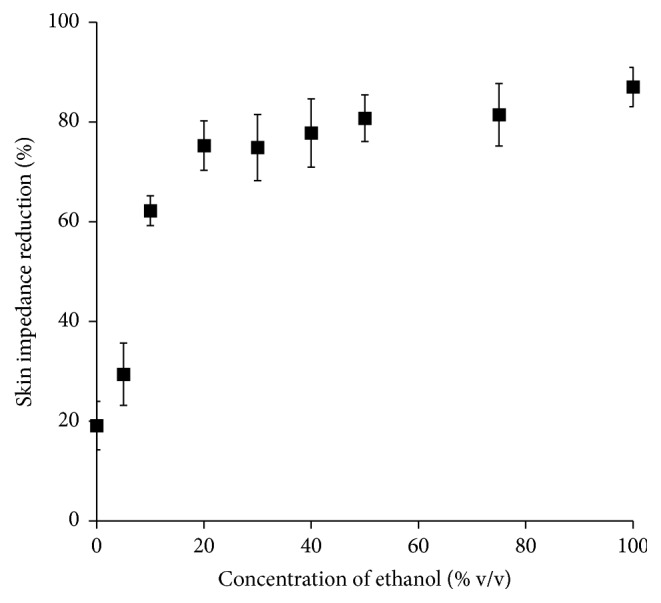
Effect of different concentrations of ethanol on the percentage of skin impedance reduction. Each value represents the mean ± SE (*n* = 3–5).

Skin impedance has been used as an index of the skin permeation rate of drugs, especially of hydrophilic drugs, because the decrease in skin impedance is related to decreased barrier function of the skin. Therefore, we determined skin impedance after pretreatment with different concentrations of ethanol.  Figure 3 shows the decrease in skin impedance with different concentrations of ethanol on excised back skin of hairless rats. Ethanol treatment changed the skin impedance even at a low concentration. Ethanol solution at a concentration of 5% reduced skin impedance by about 30%, and 20–100% ethanol could reduce skin impedance about 75–80%.

#### 3.1.3. Stratum Corneum Lipid Liposomes Characteristics

Table 2 summarizes the characteristics of blank SCLLs and FL-SCLLs prepared in the present study. The vesicle size and distribution of FL-SCLLs were a little larger but narrower, respectively, than the blank SCLLs, indicating that the vesicle dispersion was homogeneous. Both the liposome formulations showed highly negative surface charge of −87.0 ± 2.3 and −99.6 ± 3.3 mV, respectively, because of the ionized fraction of fatty acids at pH 9.0. The presence of FL anions inside the SCLLs leads to higher negative surface charge. The zeta potential of both SCLL formulations indicated high electrostatic repulsion among the particles and excellent kinetic stability [19].

#### 3.1.4. Effect of Ethanol on the Sodium Fluorescein Leakage from Stratum Corneum Lipid Liposomes

**Figure 4:**
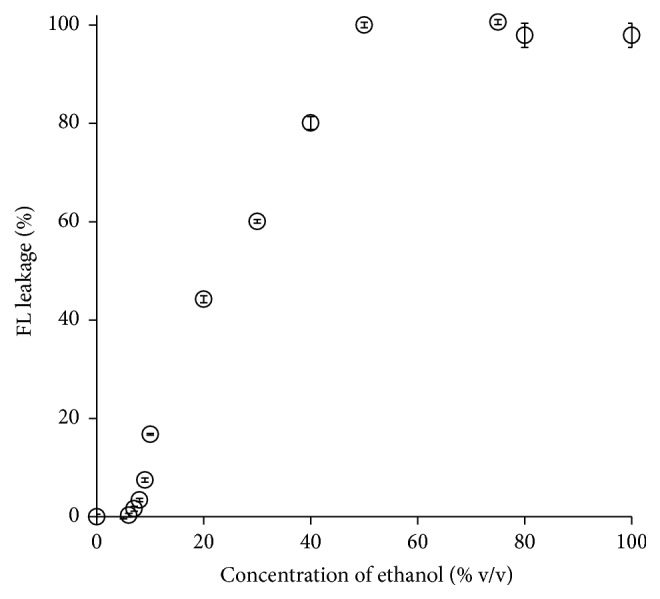
Effect of different concentrations of ethanol on the sodium fluorescein (FL) leakage from stratum corneum lipid liposomes (SCLLs). Each value represents the mean ± SE (*n* = 3).

Next, the effect of different concentrations of ethanol was tested on the disruption of SCLLs.  Figure 4 shows the relation between the FL leakage (%) and ethanol concentration. As the ethanol concentration increased, FL leakage also increased in a concentration-dependent manner. At low ethanol concentrations (0–5% ethanol), no significant promotion was observed in FL leakage compared with the control (0.1 M borate buffer). At higher concentrations of ethanol, on the other hand, FL leakage increased proportionally with the concentration. However, no more FL leakage was observed at more than 50% ethanol.

#### 3.1.5. Effect of Ethanol on Membrane Fluidity of Stratum Corneum Lipid Liposomes

**Figure 5:**
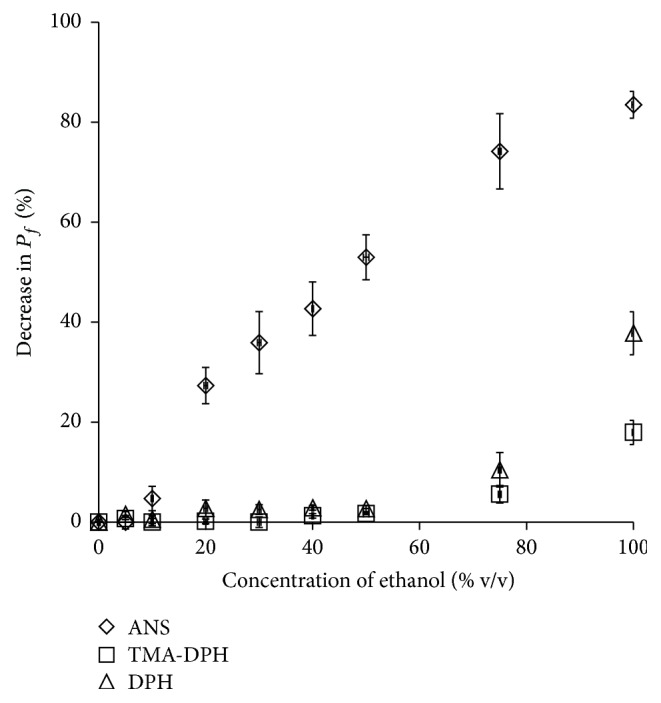
Effect of different concentrations of ethanol on the percentage decrease in fluorescence polarization (*P*_*f*_) using three fluorescent probes: 1,6-diphenyl-1,3,5-hexatriene (DPH), N,N,N-trimethyl-4-(6-phenyl-1,3,5-hexatrien-1-yl)phenyl-ammonium* p*-toluenesulfonate (TMA-DPH), and 8-anilino-1-naphthalenesulfonic acid ammonium salt (ANS). Each value represents the mean ± SE (*n* = 3).

Figure 5 shows the membrane fluidity of blank SCLLs incubated with fluorescent probes with different lipophilicities. The decrease in *P*_*f*_ was used as an index of membrane fluidity of SCLLs. The results with DPH and TMA-DPH as fluorescent probes showed relatively low membrane fluidity. The maximum decrease was observed at 100% ethanol, which provided 37.8% and 17.9% decreases in *P*_*f*_ for DPH and TMA-DPH, respectively. High membrane fluidity was observed when using ANS probe at low concentrations of ethanol.

#### 3.1.6. Relationship between Sodium Fluorescein Leakage and Increase in Skin-Penetration-Enhancement Ratio

**Figure 6:**
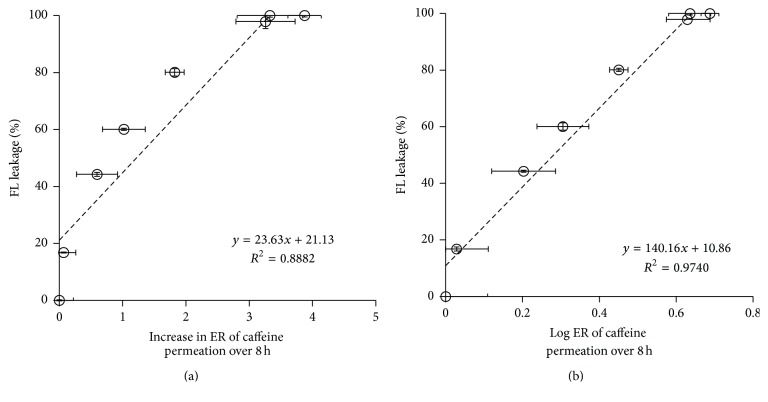
The relationship between sodium fluorescein (FL) leakage and increase in the skin penetration-enhancement ratio (ER) (a); log⁡ER of caffeine permeated over 8 h from each concentration of ethanol solution (b). Each value represents the mean ± SE (*n* = 3–5).

SCLLs were used as an SC lipid model to investigate the enhancing effect of ethanol on the skin permeation of caffeine. The correlation was evaluated between the present SCLL-based HTS data and the conventional skin-penetration-enhancing profiles of caffeine.  Figure 6(a) shows the relationship between the increase in ER of the cumulative amount of caffeine permeated through skin over 8 h (*x*-axis) and FL leakage (%) from SCLLs (*y*-axis). The correlation coefficient of this relationship was 0.888. The obtained profile was concave relative to *x*-axis, indicating that ethanol more markedly affected SCLL disruption than the skin permeation of caffeine, especially at low concentrations of ethanol. Almost linear relationship could be observed at higher concentrations of ethanol. The semilog plot, as shown in Figure 6(b), shows a very high correlation coefficient of 0.974.

#### 3.1.7. Relationship between Stratum Corneum Lipid Liposome Membrane Fluidity and Increase in Skin Penetration-Enhancement Ratio

**Figure 7:**
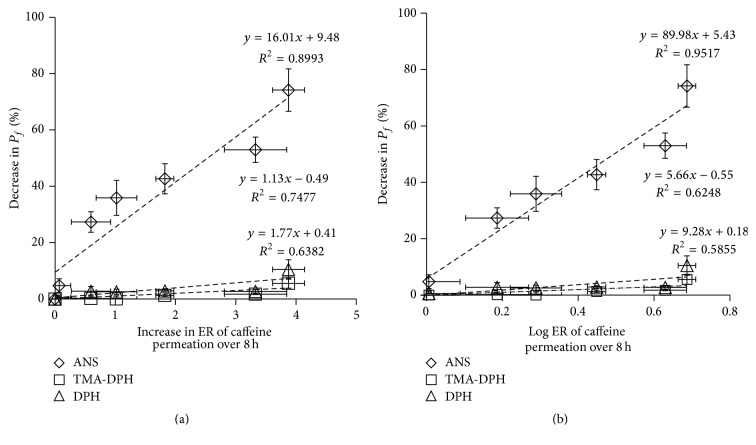
The relationship between percentage of decrease in fluorescence polarization (*P*_*f*_) using various fluorescent probes and increase in the skin penetration-enhancement ratio (ER) (a); log⁡ER of caffeine permeated over 8 h from each concentration of ethanol solution (b). Each value represents the mean ± SE (*n* = 3–5). The data from 100% ethanol was excluded in this figure.

Figure 7(a) shows a relationship between SCLL membrane fluidity and the increase in the ER. The data from 100% ethanol were not included in this correlation, because the reduction in the ER might be an exceptional case, with such a high concentration of ethanol having a reversed effect on the skin permeation of caffeine. The linear relationship could be obtained in SCLL membrane fluidity measured using the ANS probe with a correlation coefficient of 0.899, whereas DPH and TMA-DPH showed poorer correlations (*R*^2^ of DPH and TMA-DPH: 0.638 and 0.748, resp.). In the data using ANS probe, a higher correlation was obtained in the semilog plot as shown in Figure 7(b), similar to Figure 6(b).

#### 3.1.8. Relationship between Skin Impedance Reduction and Increase in Skin Penetration-Enhancement Ratio

**Figure 8:**
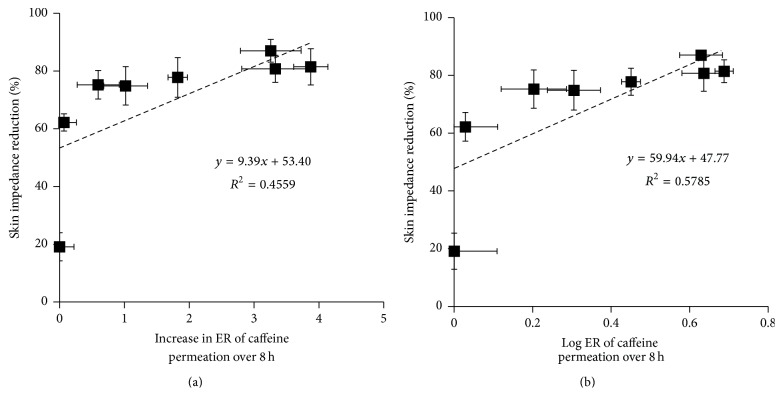
The relationship between skin impedance reduction and increase in the skin penetration-enhancement ratio (ER) (a); log⁡ER of caffeine permeated over 8 h from each concentration of ethanol solution (b). Each value represents the mean ± SE (*n* = 3–5).

Figure 8 shows a relation between skin impedance reduction and increase in ER. No good relationships were observed; the correlation coefficient was 0.456 between the increase in ER at 8 h and impedance reduction, although such skin impedance was reported to be correlated with a big amount of skin permeation data of drugs.

### 3.2. Discussion

In the present study, an HTS approach for searching for effective CPEs was established based on using SCLLs as a model membrane mimicking the primary skin-penetration domain of the SC lipid bilayer. Different concentrations of ethanol were used in the first step, because this is a well-known CPE contained in many topical and transdermal formulations. The composition of lipids used in SCLLs, closely similar to the lipid composition of intercellular lipids, was comprised of 33% ceramide type III, 22% ceramide type IV, 15% fatty acids (palmitic : lignoceric : octacosanoic acid: 1 : 2 : 1), 25% cholesterol, and 5% cholesteryl sulfate and buffer (pH, 9.0), which was reported to enhance the stability of SCLL vesicles [18]. A fluorescent hydrophilic marker, FL, was entrapped in SCLLs in order to monitor the degree of its membrane rupture by ethanol. Furthermore, SCLL membrane fluidity using probe of various lipophilicities was also measured in an HTS manner. These experiments were compared with conventional methods for the determination of skin penetration-enhancing effect of chemicals:* in vitro* permeation and impedance studies using hairless rat skin.

Skin permeation experiments with caffeine were conducted in asymmetric conditions to determine the penetration-enhancing effect of ethanol. The increase in ER by ethanol was concentration-dependent (Figures 1 and 2). A similar ethanol concentration-dependent effect was observed when using indomethacin in our previous study [20]. However, the permeation of caffeine decreased at the highest ethanol concentration, because highly concentrated ethanol dehydrates the skin membrane and reduces the skin permeation of drugs [21]. Our previous study also found that the skin permeability of hydrophilic drugs was increased but inversely decreased by low and high concentration of ethanol, respectively [22]. In addition, X-ray diffraction data confirmed that low concentrations of ethanol disturbed the short lamellar structure of SC lipids, but the high concentration caused an aligned structure [23].

Skin impedance has been reported to correlate with drug permeability through skin [22, 24]. Then, we determined the back skin impedance to increase the sensitivity of ethanol on impedance and to reduce the number of rats sacrificed. Ethanol could reduce the skin impedance and clearly alter the flux of low molecular weight ions through the skin [22]. Nevertheless, the relationship was not linear between the reduction in skin impedance and the increase in ER (Figure 8). This is because low concentrations of ethanol could greatly reduce the skin impedance but higher concentrations remained stable (Figure 3). Therefore, the reduction in skin impedance by ethanol might not be a good indicator for the skin penetration-enhancement effect. This may be because of different thermodynamic activities of caffeine in different concentrations of ethanol when using the same concentration of caffeine in different ethanol concentrations. In other words, the thermodynamic activity of a certain concentration of caffeine is low in high concentrations of ethanol, resulting in lower skin permeation of caffeine from higher concentrations of ethanol solution. Another possibility is that lower skin impedance may be obtained from the same concentration of caffeine at low ethanol concentrations because skin impedance is also dependent on the ethanol concentration in skin. When checking other CPEs rather than solvent types such as ethanol, skin impedance may be dependent on the penetration-enhancing effect, because little contribution is expected by nonsolvent type enhancers on the thermodynamic activity of penetrants.

Using SCLL leakage tests, FL leakage was observed from low (≥5%) to high (~100%) concentrations of ethanol and the degree was ethanol concentration-dependent (Figure 4), suggesting that the disruption effect on the SCLL membrane was closely related to ethanol concentration. Ethanol has been reported as a markedly effective skin penetration-enhancer by various mechanisms because of an increase in drug solubility, improvement in drug partitioning into the SC, modification of thermodynamic activity of drugs, solvent (ethanol) drag effect across the skin, fluidization and extraction of SC lipids, and the structure modification of SC keratin [21, 25]. SCLLs used in the present experiment are a model representing only the SC lipids with lack of cellular proteins such as keratin [6]. Therefore, the present SCLL leakage results might not be totally predictive for all mechanisms associated with the skin penetration-enhancing effects of ethanol.

**Figure 9:**
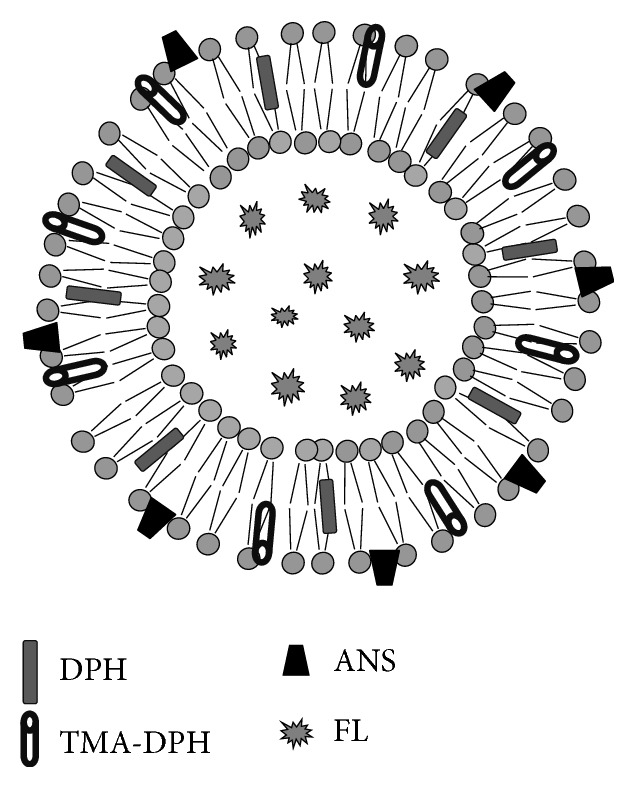
Location of fluorescent probes in stratum corneum lipid liposomes (SCLLs).

SCLLs mimic SC lipids in the intercellular domain where CPEs act. Then, three different fluorescent probes, DPH, TMA-DPH, and ANS, were used to determine the SCLL membrane fluidity in order to better understand the effect of ethanol on lipid packing. High fluidity in the SCLL membrane represents a high degree of lipid molecule disorganization, which provides high permeability of drugs through the bilayer [26]. The ethanol effect on the fluidity of SCLL was determined, especially in the region where each fluorescent probe was contained, as depicted in Figure 9. DPH and TMA-DPH serve as markers for molecular movement in the hydrophobic core and superficial surface of SCLLs [27], respectively. The effect of ethanol on SCLL fluidity using DPH and TMA-DPH showed similar patterns (Figure 5). Although lower ethanol concentrations could not promote SCLL fluidity, the effect was clearly found with more than 50% ethanol. On the other hand, ANS, a marker of molecular movement on the exterior membrane surface [27], changed membrane fluidity even at low ethanol concentrations, and good linearity was observed between SCLL fluidity and ethanol concentration (Figure 5). The results using these three fluorescent probes provided a possible effect of ethanol on SCLL membranes, suggesting that ethanol disrupted mainly the polar head region outside the liposome membrane similar to a previous report [28]. The use of ANS may be the most suitable for evaluating the skin penetration-enhancement effects from ethanol.

Good correlations were obtained especially in the FL leakage test (Figure 6) and SCLL fluidity detected using the ANS probe (Figure 7), indicating that SCLLs could be an optional model to investigate the skin penetration-enhancing effects of CPEs as well as their mode of actions. Furthermore, our approach was able to determine the enhancing effect by ethanol in a very short period. Thus, time-consuming skin permeation experiments, which normally take about 8 h to a few days, could be markedly reduced to only 30–60 min incubation times using SCLLs with probes and CPEs. Various samples could be simply and simultaneously evaluated using a 96-well plate fluorescence spectroscopy microplate reader. In addition, our method could avoid the use of animal membranes in comparison with typical skin permeation approaches. Despite the observation that this method was quite sensitive even at low concentrations of ethanol, which vary from the actual results of skin permeation experiments, this approach could be a practical tool to discover effective CPEs for use in topical and transdermal formulations.

## 4. Conclusions

The present HTS approach using SCLLs could be a promising model to determine the effect of ethanol on the skin permeability of drugs, because a good correlation was obtained from SCLL-based experiments and a skin permeation study. The ease of handling of the various fluorescent probes could also identify the possible mechanism of action of ethanol for enhancing skin permeation. Although skin impedance tests have been reported to correlate with skin permeability, SCLL results showed better predictability for the skin penetration-enhancement effect by ethanol, for which the primary action is on the disruption of SC intercellular lipids. The present findings could clarify the advantages of SCLLs not only for screening approaches to investigate the potential of CPEs but also for drug delivery systems.

## Figures and Tables

**Table 1:** Flux, permeability coefficient, and enhancement ratio of caffeine from different concentrations of ethanol.

Formulation	Flux (*μ*g/cm^2^/h)	Permeability coefficient (×10^−7^ cm/s)	Enhancement ratio
0% ethanol	0.81 ± 0.17	1.12 ± 0.24	1.00 ± 0.22
10% ethanol	0.88 ± 0.15	1.22 ± 0.21	1.07 ± 0.19
20% ethanol	1.52 ± 0.29	2.12 ± 0.40	1.60 ± 0.32
30% ethanol	1.95 ± 0.35	2.71 ± 0.49	2.02 ± 0.34
40% ethanol	2.94 ± 0.19	4.08 ± 0.26	2.82 ± 0.15
50% ethanol	3.82 ± 0.40	5.31 ± 0.55	4.33 ± 0.52
75% ethanol	3.67 ± 0.13	5.10 ± 0.19	4.85 ± 0.19
100% ethanol	4.22 ± 0.62	5.86 ± 0.86	4.26 ± 0.47

**Table 2:** Characteristics of blank stratum corneum lipid liposomes (SCLLs) and sodium fluorescein entrapped in SCLLs (FL-SCLLs).

	Blank SCLLs	FL-SCLLs
Size (nm)	221.1 ± 8.6	282.8 ± 3.2
Polydispersity index	0.556 ± 0.017	0.215 ± 0.014
Zeta potential (mV)	−87.0 ± 2.3	−99.6 ± 3.3
